# Rapid discrimination of human oesophageal squamous cell carcinoma by mass spectrometry based on differences in amino acid metabolism

**DOI:** 10.1038/s41598-017-03375-8

**Published:** 2017-06-16

**Authors:** Zhang Jianyong, Xu Jianjun, Ouyang Yongzhong, Liu Junwen, Lu Haiyan, Yu Dongliang, Peng Jinhua, Xiong Junwen, Chen Huanwen, Wei Yiping

**Affiliations:** 1 Department of Cardiothoracic Surgery, the Second Affiliated Hospital of Nanchang University, Nanchang, Jiangxi Province 330006 P. R. China; 2 Jiangxi Key Laboratory for Mass Spectrometry and Instrumentation, East China University of Technology, Nanchang, Jiangxi Province 330013 P. R. China; 3 School of Chemistry, Biology and Materials Science, East China University of Technology, Nanchang, Jiangxi Province 330013 P. R. China

## Abstract

Oesophageal cancer (OC) is associated with high morbidity and mortality, and surgery is the most effective approach to treat it. In order to reduce surgical risks and duration of surgery, we explored a new strategy to determine tumour margins in surgery. In this study, we included 128 cancerous and 128 noncancerous database entries obtained from 32 human patients. Using internal extractive electrospray ionization-MS, in positive ion detection mode, the relative abundances of *m/z* 104.13, *m/z* 116.10, *m/z* 132.13, and *m/z* 175.13 were higher in cancer tissue while the relative abundances of *m/z* 82.99, *m/z* 133.11, *m/z* 147.08, *m/z* 154.06, and *m/z* 188.05 were higher in normal tissue. Using partial least squares analysis, the mass spectra of cancer samples was discriminated from those of normal tissues, and the discriminatory ions were obtained from loading plots. Dimethylglycine(*m/z* 104), proline(*m/z* 116), isoleucine(*m/z* 132), asparagine(*m/z* 133), glutamine(*m/z* 147), and arginine(*m/z* 175) were identified by collision-induced dissociation experiments. Using the ROC curve analysis, we verified the validity of six amino acids for the identification of tumour tissue. Further investigations of tissue amino acids may allow us to better understand the underlying mechanisms involved in OC and develop novel means to identify tumour tissue during operation.

## Introduction

Oesophageal cancer (OC) is one of the most common types of cancer and the sixth leading cause of cancer-related mortality^1^. Oesophageal squamous cell cancer (OSCC) has been reported to be the predominant histological type of OC in China^2^. Surgery is the most effective way to cure OSCC^3^. While incomplete excision leads to local recurrence, excessive resection can lead to complications such as anastomotic leakage, recurrent laryngeal nerve injury, dysplasia, and reflux^4^. Therefore, it is vital to identify precise tumour margins during the surgery to obtain confident resection and accurate prognosis, as well as to minimize losses to healthy tissues^5^. Tumour margins can be preoperatively determined via medical imaging approaches such as chest radiography, barium meal examination of the upper gastrointestinal tract, computerized tomography, esophagogastroduodenoscopy (EGS), positron emission tomography, and endoscopic ultrasonography (EUS)^6^. Tumours are excised within a predefined safety zone or ‘resection margin’ which is defined by the size, location, and stability of the tumour. Currently, tumour margins are accurately determined intraoperatively by frozen-section histology, which is the gold standard method at present^7^; however, it is associated with many drawbacks: its time-consuming (30–40 min) nature considerably lengthens the exposure of the patient to the general anaesthetic and operative risk, and the diagnosis with this procedure is subjective (the reliability and precision of cancer diagnosis largely depend on the skills and experience of each doctor)^5, 8, 9^. Therefore, a new diagnostic method is urgently needed that could provide real-time, *in situ* identification of arbitrary tissue intraoperatively.

Metabolites are the end products of cellular regulatory processes, and metabolite levels are the ultimate response of other omics to environmental changes. Using metabolites as biomarkers and diagnostic markers of disease is desirable because they can be measured quantitatively and comprehensively^10–12^. Thus, improved understanding of the molecular mechanism involved in tumour metabolic reprogramming may assist in the discovery of new molecular diagnostic methods to identify tumour margins. Amino acids and small molecules play an important role in biological processes because they are extensively involved in metabolism^10^. Amino acids are of increasing interest in the field of metabolomics, which aims to establish the metabolic responses of living systems to external or internal perturbations. Studies have reported that glutamine could be an energy source for proliferating cancer cells^13^. Amino acid profiles, including glutamic acid, histidine, proline, and tyrosine, have also been used to predict the recurrence of breast cancer before clinical diagnosis^14^. Amino acids and small molecules play an important role in cancer metabolic pathways, but traditional methods focus on protein and nucleic acids rather than amino acids and small molecules. Therefore, in the current study, we used a novel strategy to distinguish tumour tissue on the basis of trace difference in amino acids and small molecules.

Mass spectroscopy (MS) has many advantages, including speed of analysis, high sensitivity, low limit of detection, and lack of requirement for analyte-specific reagents, and is a powerful method to analyse complex mixtures^15^. To overcome the shortcomings of frozen-section histology, a series of analytical methods have been gradually developed and established. These include desorption electrospray ionization-mass spectrometry imaging (DESI-MSI)^16–18^, matrix-assisted laser desorption ionization-mass spectrometry imaging (MALDI-MSI)^19–21^, rapid evaporative ionization-mass spectrometry (REIMS)^6, 7, 22^, and tip-spray ionization-mass spectrometry (TSI-MS)^23^. Although DESI-MSI and MALDI-MSI are more efficient than frozen-section histology, they are time-consuming methods due to the sampling, sectioning, and imaging processes. REIMS is relatively rapid as surgical removal of tissue and mass spectrometric sampling are performed nearly simultaneously; however, as tissue is destroyed in REIMS, data are harder to correlate with traditional histopathological analysis results, which is the gold standard for diagnosis^6, 7, 22^. In TSI-MS for tissue assay, the signals are intermittent and could only last several seconds. Internal extractive electrospray ionization-MS (iEESI-MS)^24–27^, which can provide molecular information within a bulk volume with high efficiency, allows both qualitative and quantitative analysis of analytes distributed in a three-dimensional volume of a range of biological tissues (e.g., leaves, fruits, roots, or lung tissues) without pretreatment^25^. An advantage of iEESI-MS is that the analysed tissue can be evaluated by histopathology.

In this study, we analysed trace differences in the metabolism of amino acids and small molecules to distinguish between OC tissue and adjacent matched normal tissue samples obtained by iEESI-MS. Then, the mass spectra of tissues were classified using partial least squares analysis (PLS). Key amino acids and small molecules with the most influence on the separation between sample classes were identified, and their chemical structures were studied by collision-induced dissociation (CID). This novel technique to intraoperatively predict tumour margins was associated with reduced operation risk and time.

## Results

### iEESI-MS of oesophageal tissue

**Figure 1:**
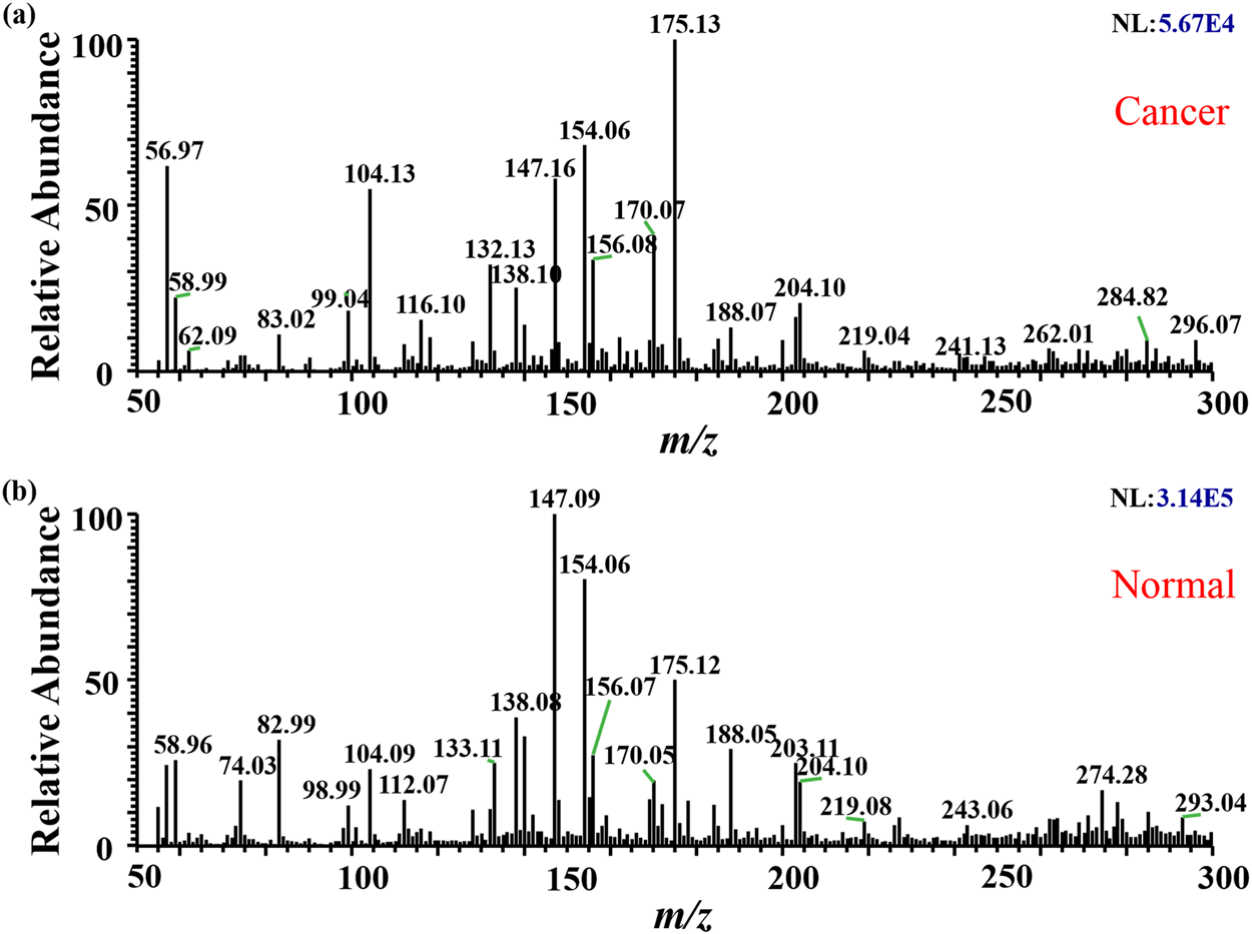
Spectra obtained from human oesophageal cancer tissues and adjacent normal tissue. (**a**) Cancerous tissue in positive ion mode with main peaks at m/z 56.97, m/z 104.13, m/z 116.10, m/z 132.13, m/z 147.16, m/z 154.06, m/z 156.08, and m/z 175.13. (**b**) Normal tissue in positive ion mode with main peaks at m/z 58.96, m/z 74.03, m/z 82.99, m/z 133.11, m/z 138.03, m/z 147.09, m/z 154.06, m/z 175.12 and m/z 188.05.

The signals are caught near real-time when the ionized chemicals enter the mass spectrometer. In the positive ion mode, the mass range was set at 50–300 Da, the main peaks of the cancer tissues were *m/z* 56.97, *m/z* 104.13, *m/z* 116.10, *m/z* 132.13, *m/z* 147.16, *m/z* 154.06, *m/z* 156.08, and *m/z* 175.13 while those of the normal tissues were *m/z* 58.96, *m/z* 74.03, *m/z* 82.99, *m/z* 133.11, *m/z* 138.03, *m/z* 147.09, *m/z* 154.06, *m/z* 175.12, and *m/z* 188.05 (Fig. 1). In cancerous tissue, the relative abundances of *m/z* 104.13, *m/z* 116.10, *m/z* 132.13, and *m/z* 175.13 were increased, while the relative abundances of *m/z* 82.99, *m/z* 133.11, *m/z* 147.08, *m/z* 154.06, and *m/z* 188.05 were decreased (Table 1). The analysis can last several minutes stably and the relative intensities were found to be highly reproducible (Table S1,2). This experiment was also repeated using samples from different extractions of the same tissue. The variation in the total ion intensity was still within one order of magnitude, with similar relative intensities obtained for each analysis.

**Table 1:** Comparison of relative abundances between cancer tissues and normal tissue.

Ions	Cancer	Normal
*m/z* 82.99	1.08 ± 0.05	4.60 ± 2.14
*m/z* 104.13	5.40 ± 0.85	2.39 ± 1.53
*m/z* 116.10	1.59 ± 0.15	0.94 ± 0.02
*m/z* 132.13	3.75 ± 0.34	2.38 ± 1.25
*m/z* 133.11	1.68 ± 1.41	2.20 ± 0.60
*m/z* 147.08	6.57 ± 0.91	7.97 ± 3.68
*m/z* 154.06	3.58 ± 0.63	5.86 ± 2.74
*m/z* 175.13	9.85 ± 0.86	6.80 ± 1.35
*m/z* 188.05	3.39 ± 1.54	4.67 ± 1.26

In cancerous tissue, the relative abundance values of *m/z* 104.13, *m/z* 116.10, *m/z* 132.13, and *m/z*175.13 were increased, while the relative abundance values of *m/z* 82.99, *m/z* 133.11, *m/z*147.08, *m/z* 154.06, and *m/z* 188.05 were decreased.

### Discrimination of cancerous tissue from normal tissue by partial least squares analysis (PLS)

**Figure 2:**
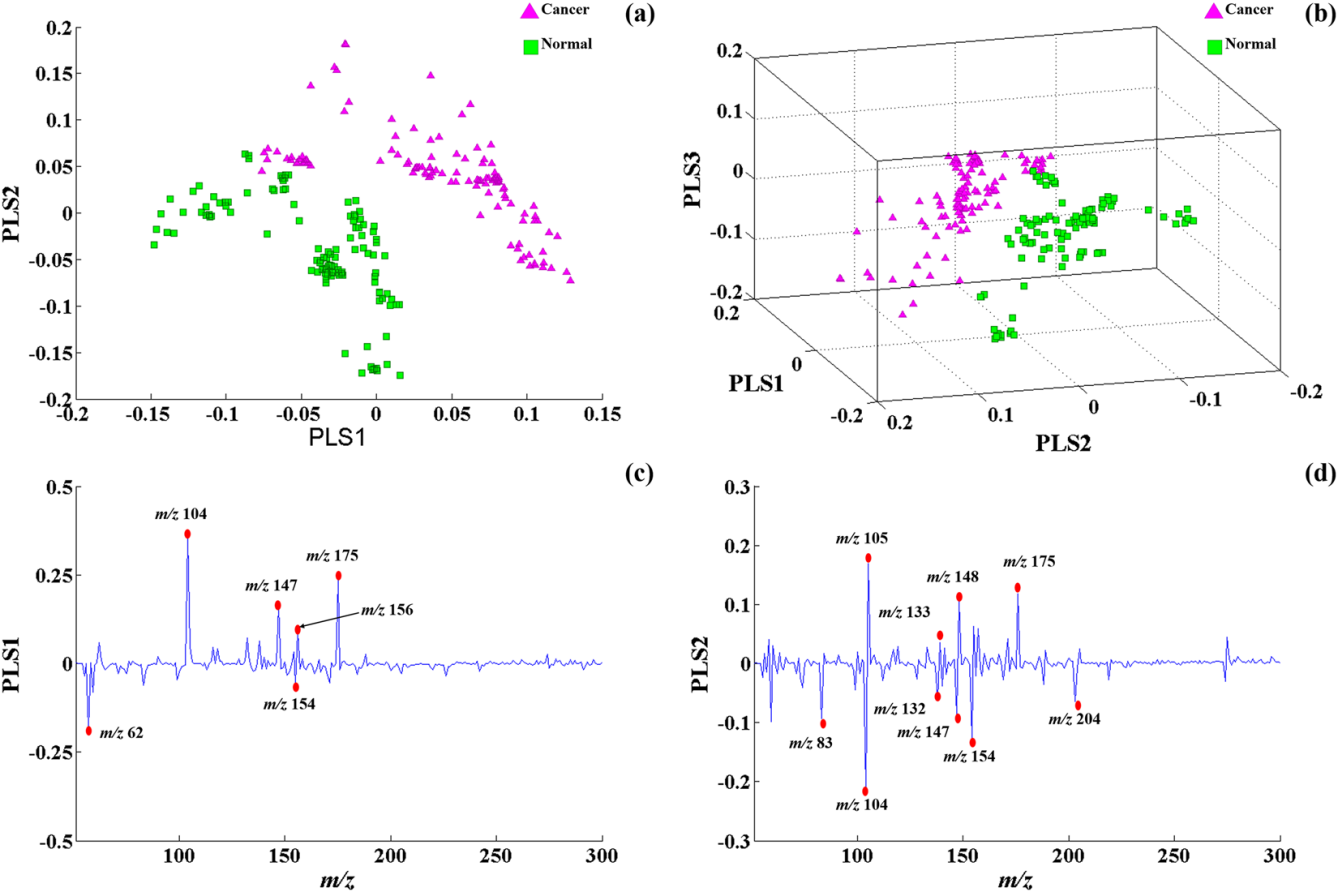
PLS analysis of oesophageal tissue mass fingerprinting. (**a**) Score plots for PLS1 and PLS2; (**b**) A 3D model to distinguish oesophageal cancer tissue from normal tissue; (**c**) Loading plots for PLS1 in PLS from the training set. (**d**) Loading plots for PLS2 in PLS from the training set. The plots illustrate the m/z values and their relative importance in the PLS analysis.

All the mass spectral data expressed by the relative abundance were directly used for the PLS. A total of 256 analysed sample database entries, including 128 cancerous samples and 128 normal tissue samples, from 32 pairs of matched samples (four points per sample) were interpreted in the score graphs of the PLS. After PLS analysis, the cancer and normal tissue could be clearly distinguished on the basis of the recorded spectra (Fig. 2a). A three-dimensional model was established for distinguishing oesophageal cancerous from normal tissue (Fig. 2b). The discriminatory ions between cancerous and normal tissue were *m/z* 62, *m/z* 83, *m/z* 104, *m/z* 105, *m/z* 132, *m/z* 133, *m/z* 147, *m/z* 148, *m/z* 154, *m/z* 156, *m/z* 175, and *m/z* 204 (Fig. 2c and d).

### Discriminatory ions identified by CID experiments

**Figure 3:**
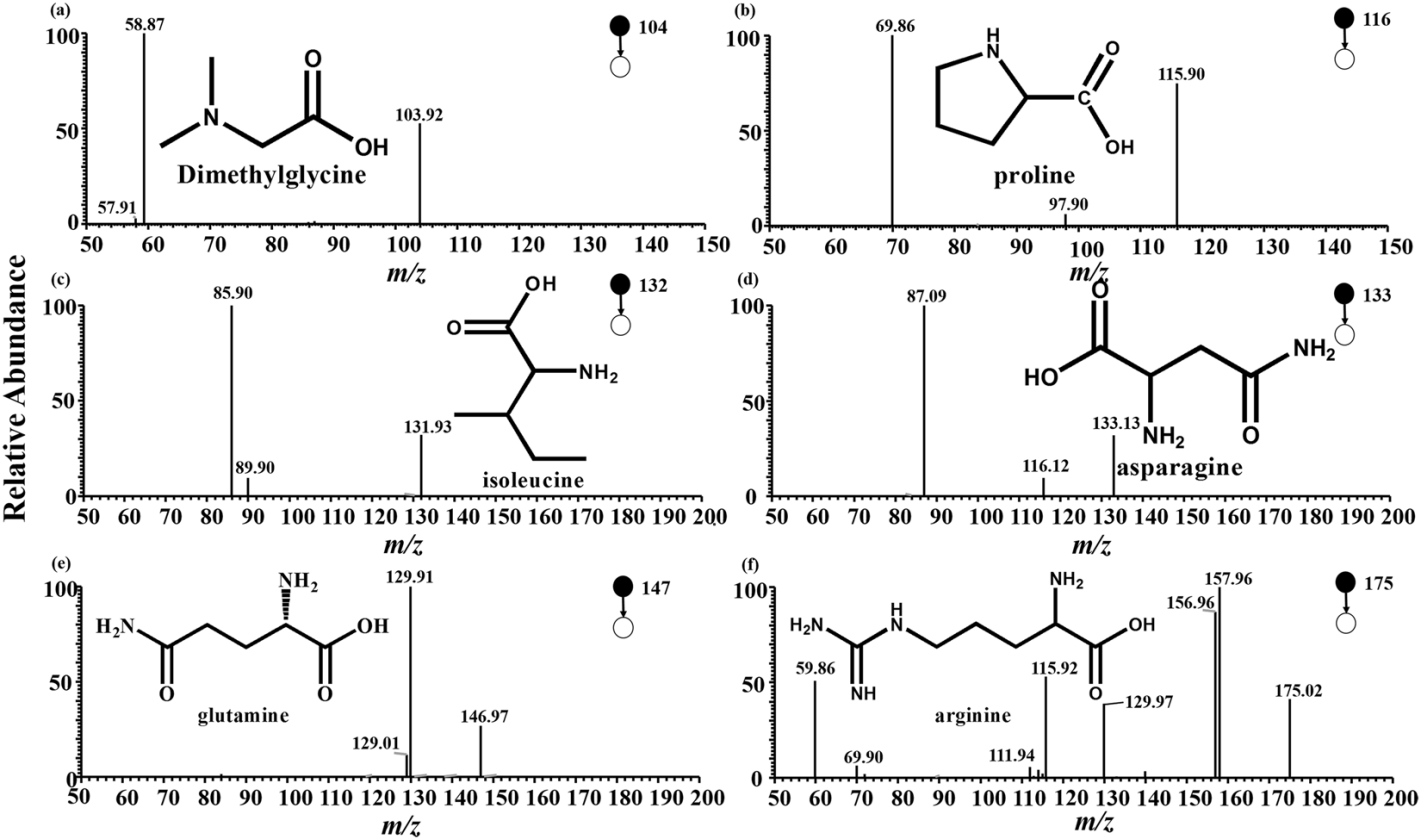
Product ion mass spectra by collision-induced dissociation: (**a**) DMG + H + (precursor ion m/z 104.13); (**b**) proline + H + (precursor ion m/z 116.10); (**c**) isoleucine + H + (precursor ion m/z 132.13); (**d**) asparagine + H + (precursor ion m/z 133.11); (**e**) glutamine + H + (precursor ion m/z 147.08), and (**f**) arginine + H + (precursor ion m/z 175.13).

Six amino acids were tentatively identified on the basis of the CID experiments, and these were compared with standard product CID data. The analytes with *m/z* 104.13, *m/z* 116.10, *m/z* 132.13, *m/z* 133.11, *m/z* 147.08, and *m/z* 175.13 were dimethylglycine (DMG), proline, isoleucine, asparagine, glutamine, and arginine, respectively (Fig. 3).

### Assessment performance of six amino acids to determine tumour margins by receiver operating characteristic (ROC) curve

**Figure 4:**
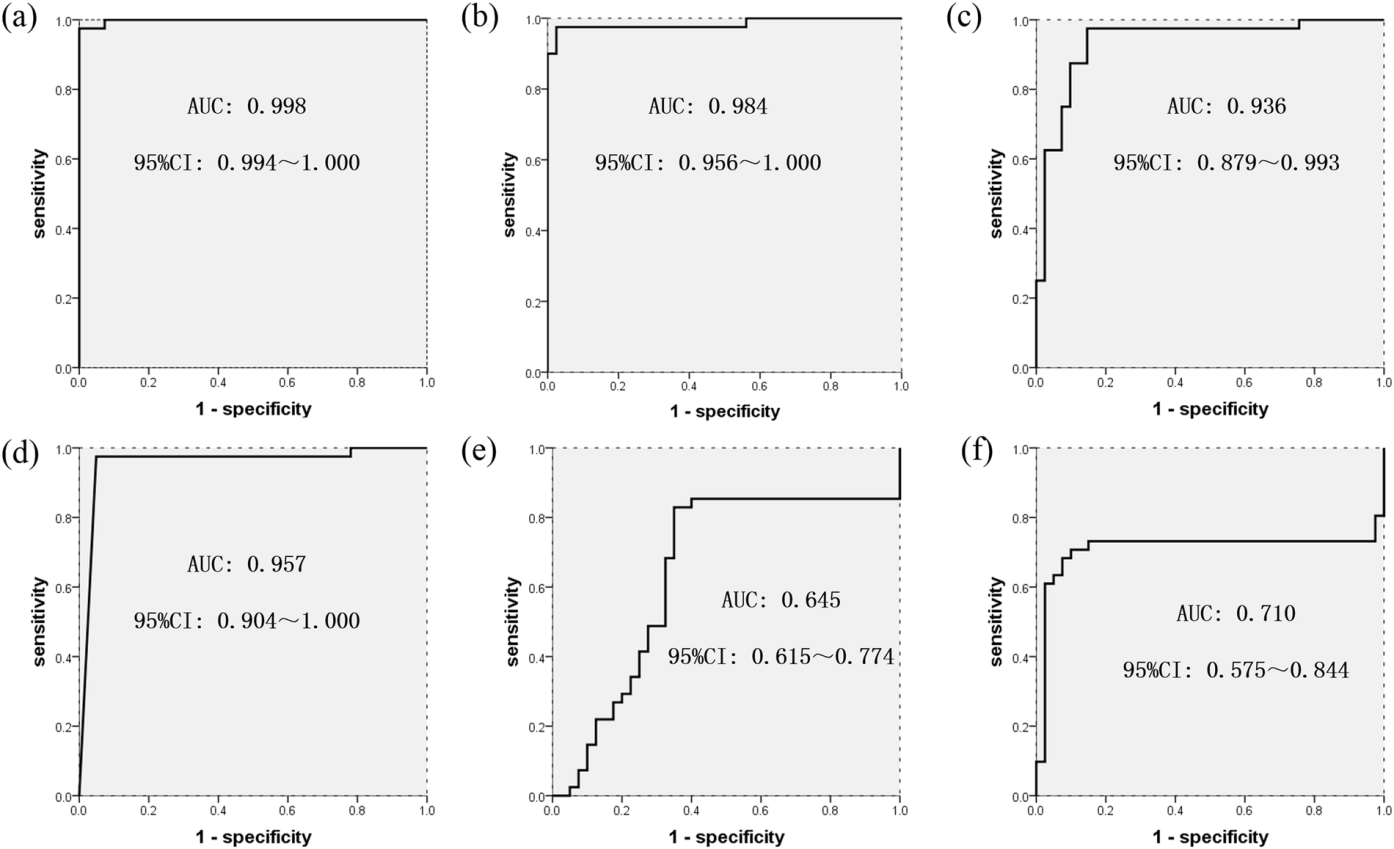
ROC curves of six amino acids; (**a**–**f**) the AUC and 95% CI of dimethylglycine (DMG), proline, isoleucine, arginine, asparagine, and glutamine, respectively; *AUC, area under the receiver operating characteristic (ROC) curve; 95% CI, 95% confidence interval.

The ROC curve, which is defined as a plot of test sensitivity as the Y coordinate versus its 1-specificity as the X coordinate, is an effective method of evaluating the quality or performance of diagnostic tests, and is widely used in clinical medicine to evaluate the performance of many clinical tests^28^. Rigorous statistical assessment of a new diagnostic method to segregate tumour margin from normal tissue is a prerequisite before it can applied in clinical medicine. ROC analysis is used to evaluate the abilities of biological markers to differentiate between the presence or absence of a disease^28^. In our present study, in the ROC curve analysis to distinguish between cancer and normal tissues, the areas under the curves (AUC) for dimethylglycine (DMG), proline, isoleucine, arginine, asparagine, and glutamine were 0.998 (95% confidence interval [CI], 0.994–1.000), 0.984 (95% CI, 0.956–1.000), 0.936 (95% CI, 0.879–0.993), 0.957 (95% CI, 0.904–1.000), 0.645 (95% CI, 0.615–0.774) and 0.710 (95% CI, 0.575–0.844), respectively (Fig. 4 and Table 2).

**Table 2:** The AUC and 95% CI of amino acids by ROC curve analysis.

Ions	Name	AUC	95%CI
*m/z* 104.13	Dimethylglycine	0.998	0.994–1.000
*m/z* 116.10	Proline	0.984	0.956–1.000
*m/z* 132.13	Isoleucine	0.936	0.879–0.993
*m/z* 133.11	Asparagine	0.645	0.615–0.774
*m/z* 147.08	Glutamine	0.71	0.575–0.844
*m/z* 175.13	Arginine	0.957	0.904–1.000

AUC, area under the receiver operating characteristic (ROC) curve; 95% CI, 95% confidence interval.

## Discussion

*In situ* and near real-time identification of the tumour margins is very important to ensure radical tumour resection and to minimize loss of healthy tissue^5, 8^. Currently, tumour margins are established preoperatively by medical imaging and intraoperatively by frozen-section histology. Although frozen-section analysis is the gold standard for tumour diagnosis and decision making regarding excision, it still has many shortcomings. First, histological methods cannot provide instant results. Traditionally, histological analysis involves fixation, embedding, staining, and sectioning, which usually takes at least 30–40 minutes for diagnosis while the patient remains under surgical anaesthesia. This process would prolong the operation duration and increase the risk of surgical exposure. Second, another problem is subjective interpretation of the results. The pathologist make a pathological diagnosis based on the visual perception of morphological features, which is extremely subjective^5, 8, 9^.

MS is used for tissue analysis, and an important advantage of MS is the objectivity of information. Tissue analysis by MS during surgery may be a new alternative to standard frozen-section histology. There have been several recent advances in MS, and a number of ionization methods: including desorption electrospray ionization-mass spectrometry imaging (DESI-MSI)^29–31^, matrix-assisted laser desorption ionization-mass spectrometry imaging (MALDI-MSI)^32^, rapid evaporative ionization-mass spectrometry (REIMS)^8^, and tip-spray ionization-mass spectrometry (TSI-MS)^23^ have enabled tissue characterization without chemical extraction. However, Although DESI-MSI and MALDI-MSI analyses can be done in a shorter time than frozen-section histology, these MS approaches still require a period of time to obtain whole-tissue images, and a certain portion of the tissue needs to be cut for MSI analysis. REIMS identifies tissue by analysis of the aerosol released from tissue, however, some molecular information may be lost with this approach. In our earlier study, we used TSI-MS to directly analyse tissue but due to lack of stability, the analysis only lasted several seconds. Therefore, a rapid and accurate approach for distinguish cancer tissue from normal tissue was necessary. iEESI-MS is an ambient mass spectrometry technique with high extraction efficiency and stability, and our group has successfully used this technique *in situ* and with the ability to obtain near real-time results of biological tissue, urine, and bulk samples^26, 27, 33, 34^. In iEESI, extraction solution charged at a high voltage is directly infused into the three-dimensional volume of the analysed sample through the inserted capillary. The analytes are then extracted by the infused solvent and carried along the electric field gradient inside the bulk volume of the sample^25^.

In this study, we used iEESI-MS to successfully identify differential metabolism of certain amino acids between cancer and normal (control) tissue. In the positive ion mode, the relative abundance values of dimethylglycine, proline, isoleucine, and arginine were higher in cancer tissue than in normal tissue, while the relative abundances of asparagine and glutamine, which were clearly lower in the adjacent normal tissue. This is a novel strategy of studying the metabolism of amino acids and small molecules in malignant tumours. We found that many studies have investigated the association between amino acid metabolism and cancer. For instance, proline can affect oncogenes or suppressor genes^11^; glycine and serine are essential for tumour growth^35^; The change in arginine metabolism evidenced by reduced plasma arginine (ARG) concentrations and arginine restriction inhibit cell migration, and have been found in various types of cancer^36, 37^. Cancer cells use glucose and glutamine as the major sources of energy and precursor intermediates, and enhanced glycolysis and glutaminolysis are the major hallmarks of metabolic reprogramming in cancer^38^. In our study, the glutamine level was clearly lower in normal tissue, probably because the cancer cells were more capable of storing glutamine or increasing glutamine uptake.

We used iEESI-MS to analyse OC tissue and adjacent normal tissue and used PLS to distinguish between the two types of tissue based on trace differences in the metabolism of amino acids and small molecules. After analysis of PLS, the recorded spectra could clearly distinguished between the cancer tissue and the normal tissue. The AUCs and the corresponding 95% CIs for each amino acid were obtained for the discovery and validation sets. To put these results in context, in a diagnostic/prognostic test, AUC values of 0.5–0.7 represent low accuracy, 0.7–0.9 represent moderate accuracy, and values > 0.9 represent high accuracy^39^. The AUC values of dimethylglycine, proline, isoleucine, arginine, asparagine, and glutamine were 0.998, 0.984, 0.936, 0.957, 0.645, and 0.710, respectively, which represent high accuracy to distinguish cancer tissue from normal tissue. Dimethylglycine, proline, isoleucine, and arginine can serve as useful potential biomarkers for *in situ* and near real-time identification of tumour margins. The meaning of the term “confidence interval” is that if the CI is constructed across many separate data analyses of replicated experiments, the proportion of such intervals that contain the true value of the parameter will match the given confidence level^40^. Two-sided confidence limits form a CI, and their one-sided counterparts are referred to as lower/upper confidence bounds (or limits). The 95% CIs of dimethylglycine, proline, isoleucine, and arginine were 0.994–1.000, 0.956–1.000, 0.879–0.993, and 0.904–1.000, respectively, suggesting that these four amino acids can serve as reliable and useful potential biomarkers for the identification of tumour margins.

Unlike normal differentiated cells, which primarily rely on mitochondrial oxidative phosphorylation to generate the energy needed for cellular processes, most cancer cells rely on aerobic glycolysis, a phenomenon termed “the Warburg effect”^41^. The metabolism of proline, asparagine, glutamine, and arginine is closely related to the citrate (TCA) cycle, which is affected by the altered energy metabolism in cancer cells^41^. In this study, we provide evidence of the significant differences in amino acid concentrations between oesophageal squamous cell carcinoma and normal tissues: while the concentrations of DMG, proline, isoleucine, and arginine were increased, those of asparagine and glutamine were decreased in oesophageal squamous cell carcinoma tissue relative to normal tissue. The altered amino acid levels in cancerous tissue may be related to “the Warburg effect.” More research is required to confirm whether a better understanding of the mechanistic links between altered amino acid metabolism and tumour growth control may ultimately lead to better treatments for human cancer.

In conclusion, we showed that iEESI-MS in combination with PLS can be used to successfully identify tumour tissue *in situ* and near real-time. Furthermore, we found that dimethylglycine, proline, isoleucine, asparagine, isoleucine, and arginine may be useful molecular biomarkers associated with the development of EC. Further investigations of tissue amino acids may allow us to better understand the underlying mechanisms involved in OC and to develop novel means to identify tumour tissues in operation.

## Materials and Methods

### Sample collection

This study was approved by the Medical Ethics Committee of the Institutional Review Board of the Second Affiliated Hospital to Nanchang University, Nanchang, P. R. China. Written informed consent was obtained from all the patients in this study. All clinical investigations were conducted according to the principles expressed in the Declaration of Helsinki. We enrolled 32 patients with OSCC and their diagnosis was confirmed by pathological analysis. Table 3 presents the patient demographic characteristics and histological classification of the cases. The patients had no other oesophageal diseases, accompanying malignancies, and no history of preoperative chemotherapy or radiotherapy. Samples from each patient consisted of OC tissue and matched normal tissue (obtained 5 cm from the tumour margin). The tissue samples were collected in liquid nitrogen within 5 min by a trained surgeon and then stored at −80 °C.

**Table 3:** Patient demographics and clinical information.

Male/female	26/6
Median age (range)	62.5 (37–84)
Location	
Middle thoracic	22
Lower thoracic	10
Degree of differentiation	
Well	2
Moderate	19
Poorly	11
T stage	
T 1	9
T 2	14
T 3	8
T 4	1
N stage	
N 0	21
N 1	10
N 2	1
N 3	0
M stage	
M 0	32
M 1	0

### iEESI-MS

**Figure 5:**
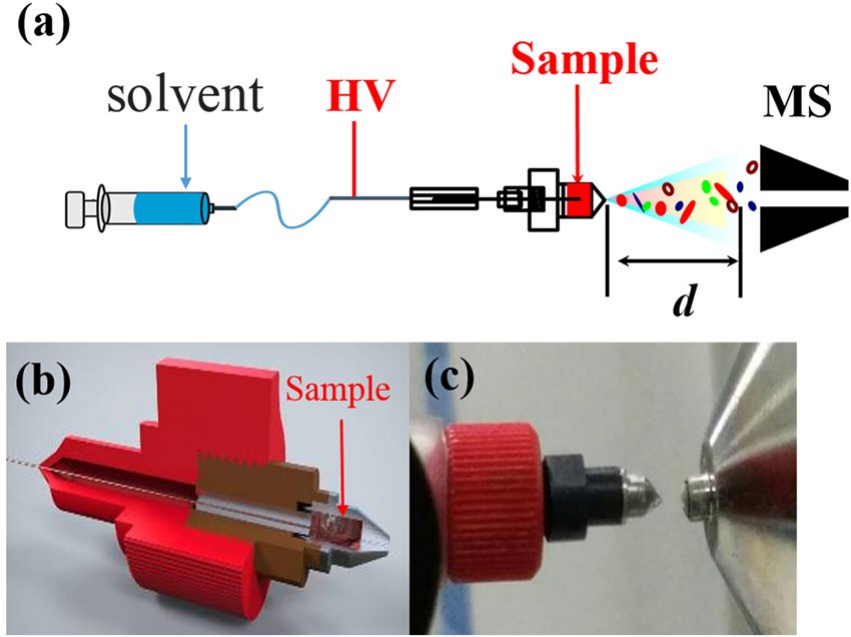
The iEESI-MS source. (**a**) Schematic diagram of iEESI-MS: Signals of the interior analytes were captured when the solvent was delivered from an infusion pump at a flow rate 0.5 μL·min^−1^ and supplied with a high voltage +4.5 kV. (**b**) Cross-sectional drawing of the iEESI-MS source. (**c**) Physical map source of iEESI-MS. HV is high voltage.

A small (approximately 1 mm3) piece of oesophageal tissue (designated the analysis sample) was cut using a disposable sterilized surgical blade (Cardinal Health, Dublin, OH, USA), and directly loaded into groove of the iEESI-MS source (Fig. 5). A Taylor cone is formed on the tissue when placed in solvent and subjected to high voltage. The internal chemicals of the tissue sample were extracted and continuously charged by the ionizing solvent (methanol/water/acetic acid, 50:50:0.01 v/v/v), which was delivered via an infusion pump at a flow rate 0.5 μL·min^−1^ and subjected to a high voltage of + 4.5 kV (Fig. 5). All MS spectra were collected using a linear trap quadrupole mass spectrometer (LTQ-XL, Thermo Scientific, San Jose, CA) equipped with homemade iEESI ion sources (Fig. 5). The LTQ instrument was operated in the positive ion detection mode and mass spectra were acquired in the *m/z* range of 50–300. The capillary was heated to 150 °C, the capillary voltage was 35 V, and the tube lens voltage was set at 100 V. For CID, the isolated width of the precursor ion was 1.0 Da, normalized collision energy was 13–20%, and the other parameters were set at default values of the instrument. Each normal and cancerous tissue samples was analysed four times. A total of 256 samples were analysed in this study, including 128 cancerous and 128 normal tissue samples. After MS, the tissue samples were collected and sent for histopathological examination to verify the results of the MS.

### Data analysis

Mass spectra were collected in single-stage MS, positive ion mode, and the amino acids and small molecules were in the mass range of *m/z* 50–300. PLS of the mass spectral fingerprint data was performed using Matlab (version 7.8, Mathworks, Inc., Natick, MA). The iEESI-MS data obtained from human tissues were exported into Microsoft Excel and arranged according to their *m/z* values with unit resolution as independent variables and using the relative abundance of the full scan mass (MS^1^) fingerprints as the dependent variables. The entire mass spectra data were treated as matrix X, and the rows and columns corresponded to sample cases and *m/z* value variables, respectively. All the mass spectral data expressed by relative abundance were directly used for the analysis. All mass spectral data belonging to one patient were excluded from the sample set, and a new model was calculated using the remaining data. Withheld data were projected into the new model and classified as one tissue type, performed between the unknown sample point and calculated class centres. This process was repeated for each individual patient. First, mass spectrometric data were normalized using standard normal variate transformations to correct for baseline shifts and global variation in signal intensities, cancer and normal tissue data were analysed by PLS, and the key amino acids and small molecules that had the most influence on the separation between sample classes were identified. Then, ROC curves of some key chemicals and the areas under the curve (AUC) of the ROC were calculated using SPSS version 21.0 (SPSS, Chicago, IL, USA), and then used to assess the validity of the potential small molecular biomarkers and to identify the optimized cutoff values.

## Electronic supplementary material

Table s-1 AND Table s-2
