# Supplementary material for: Rapid discrimination of human oesophageal squamous cell carcinoma by mass spectrometry based on differences in amino acid metabolism
Source: Sci Rep. 2017 Jun 16;7:3738. doi: 10.1038/s41598-017-03375-8 (PMC5473808; doi:10.1038/s41598-017-03375-8)
Supplement: Supplementary file 1 — Table s-1 AND Table s-2 [file 41598_2017_3375_MOESM1_ESM.pdf]

Rapid discrimination of human oesophageal squamous cell carcinoma by mass spectrometry based on differences in amino acid metabolism

Zhang Jianyong[1,2], Xu Jianjun[1], Ouyang Yongzhong[3], Liu Junwen[3], Lu Haiyan[2], Yu Dongliang[1], Jinhua Peng [1], Xiong Jianwen[1], Chen Huanwen[2], Wei Yiping[1]

[1]Department of Cardiothoracic Surgery, the Second Affiliated Hospital of Nanchang University, Nanchang, Jiangxi Province 330006, P. R. China.

[2]Jiangxi Key Laboratory for Mass Spectrometry and Instrumentation, East China University of Technology, Nanchang, Jiangxi Province 330013, P. R. China.

[3]School of Chemistry, Biology and Materials Science，East China University of Technology, Nanchang, Jiangxi Province 330013, P. R. China.

Table s-1 The reproducibility of one sample

m/z	Intensity				Average	SD	RSD(%)
**147**	44962	42403	41572	42494	**42857.75**	1462.9	**3.41**
**154**	24680	23445	22802	23339	**23566.5**	793.9	**3.36**
**175**	18528	17467	17007	17532	**17633.5**	640.5	**3.63**

The reproducibility of the assay on replicate samplings of the one sample analysis was studied. The relative standard deviation (RSD) was obtained by measuring the abundance of ions at *m/z* 147, 154 and 175 of the normal tissue. For example, the RSD (n=4) of the three ions obtained from the patient 587104 were 3.41%, 3.36% and 3.63%, respectively.

Table s-2 The reproducibility of four sample from one patient

m/z	Intensity				Average	SD	RSD(%)
**147**	33704	29869	32446	44335	**35088.5**	6367.6	**18.14**
**154**	38860	40088	37208	50104	**41565**	5813.7	**13.99**
**175**	57304	60592	53492	72050	**60859.5**	8004.6	**13.15**

The reproducibility of the assay on replicate samplings of the four sample analysis from one patient was studied. The relative standard deviation (RSD) was obtained by measuring the abundance of ions at *m/z* 147, 154 and 175 of the cancerous tissue. For example, the RSD (n=4) of the three ions obtained from the patient 586375 were 18.14%, 13.99% and 13.15%, respectively.

The result by ESI-MS analysis

Let approximately 50–70 mg of oesophageal tissue soak in 1 ml of ultrapure water, place it at room temperature for 4 hours, and then the soaking solution was centrifuged for 5 min at 4000rpm, after discarding the pellet, the supernatant was analyzed by ESI-MS.

ESI-MS and iEESI-MS could identify the same amino acid markers. Using ESI-

MS, the relative abundances of *m/z* 104.15 was increased in the solution of normal tissue compare with the cancerous solution, it was contrary to the results of the original study.

In the positive ion mode, the mass range was set at 50-300 Da, the main peaks of the soaking solution of cancerous tissue were *m/z* 57.06, *m/z* 59.07, *m/z* 97.04, *m/z* 104.17, *m/z* 132.15, *m/z* 147.16, *m/z* 154.11, *m/z* 170.09, *m/z* 175.12, *m/z* 204.11, and *m/z* 274.33, while *m/z* 57.05, *m/z* 59.06, *m/z* 81.07, *m/z* 104.15, *m/z* 147.14, *m/z* 154.11, *m/z* 170.09, *m/z* 175.14, *m/z* 203.11, and *m/z* 274.32 were the main peaks in the soaking solution of normal tissue.

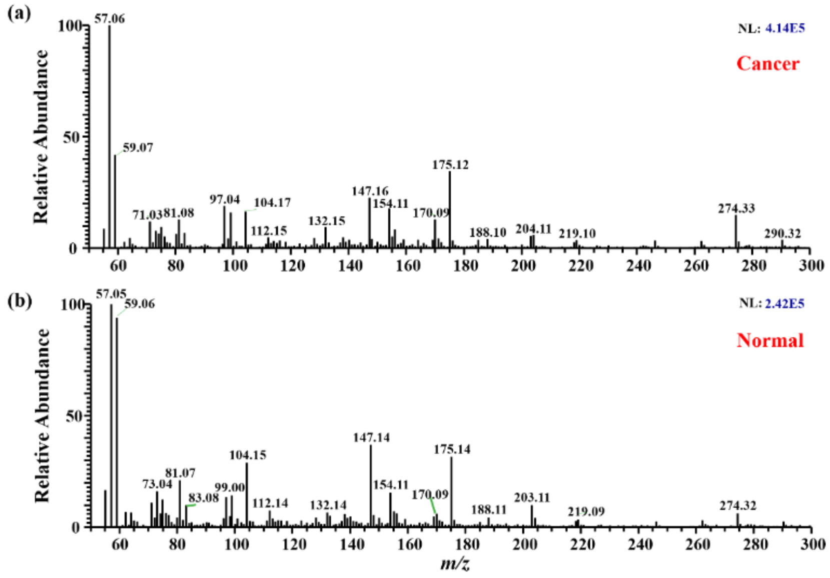

Fig. 1 Spectra obtained from the soaking solution of human oesophageal cancer tissues and adjacent normal tissue by ESI-MS. (a) the soaking solution of cancerous tissue in positive ion mode with main peaks at *m/z* 57.06, *m/z* 59.07, *m/z* 97.04, *m/z* 104.17, *m/z* 132.15, *m/z* 147.16, *m/z* 154.11, *m/z* 170.09, *m/z* 175.12, *m/z* 204.11, and *m/z* 274.33. (b) the soaking solution of normal tissue in positive ion mode with main peaks at *m/z* 57.05, *m/z* 59.06, *m/z* 81.07, *m/z* 104.15, *m/z* 147.14, *m/z* 154.11, *m/z* 170.09, *m/z* 175.14, *m/z* 203.11, and *m/z* 274.32.

Discrimination of the data of the soaking solution cancerous tissue from normal tissue by partial least squares analysis (PLS)

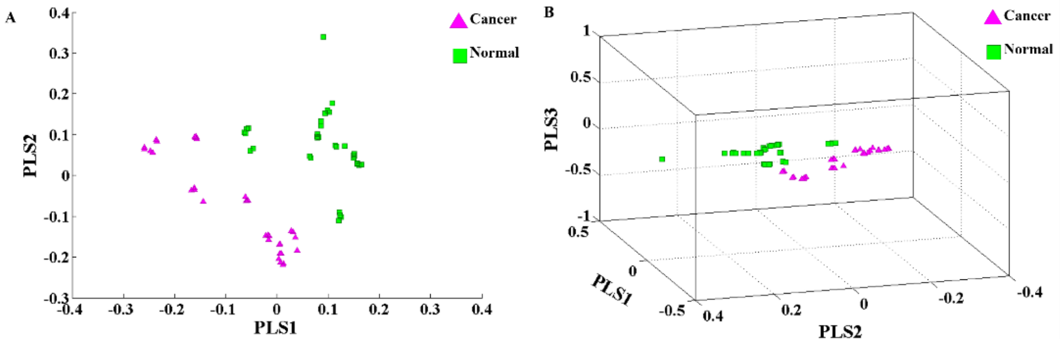

Fig. 2 PLS analysis of oesophageal tissue mass fingerprinting.

A: Score plots for PLS1 and PLS2;

B: the 3D model for discrimination oesophageal cancerous from normal tissue.
